# Patient, nurse, and physician preferences: final results of the CONVENIENCE study evaluating pegfilgrastim prophylaxis via pre-filled syringe or on-body injector in cancer patients

**DOI:** 10.1007/s00520-021-06230-9

**Published:** 2021-05-06

**Authors:** Michael Metz, Dieter Semsek, Gunther Rogmans, Ulrich Hutzschenreuter, Thomas Fietz, Johanna Harde, Stefan Zacharias, Carsten Hielscher, Andreas Lorenz, Mark-Oliver Zahn, Dagmar Guth, Steffen Liebers, Michael Berghorn, Sina Grebhardt, Christiane D. Matillon, Gerlinde Egerer, Karin Potthoff

**Affiliations:** 1 OSP Göttingen, Göttingen, Germany; 2 Praxis für interdisziplinäre Onkologie & Hämatologie, Freiburg im Breisgau, Germany; 3 ZAGO- Zentrum für ambulante gynäkologische Onkologie, Krefeld, Germany; 4 Hämatologisch-Onkologische Gemeinschaftspraxis, Nordhorn, Germany; 5 Schwerpunktpraxis für Hämatologie und Internistische Onkologie, Gastroenterologie, Singen, Germany; 6 iOMEDICO, Freiburg im Breisgau, Germany; 7 g.SUND Gynäkologie Kompetenzzentrum Stralsund, Stralsund, Germany; 8 Gynäkologische Onkologie, Frauenarztpraxis Dr. Lorenz, Hildburghausen, Germany; 9 Überörtliche Berufsausübungsgemeinschaft MVZ Onkologische Kooperation Harz, Goslar, Germany; 10 Gynäkologische Onkologie, Praxis Dr. med. Dagmar Guth, Plauen, Germany; 11 KMG Klinikum Sömmerda, Sömmerda, Germany; 12 MVZ am Allgemeinen Krankenhaus Celle, Celle, Germany; 13 Universitätsklinikum Heidelberg, Klinik Für Hämatologie, Onkologie, Rheumatologie, Heidelberg, Germany

**Keywords:** Pegfilgrastim, On-body injector, Manual injection, Patient/physician/nurse preference

## Abstract

**Purpose:**

The on-body injector (OBI) automatically delivers pegfilgrastim the day after chemotherapy (CTx), thus eliminating the need of return visits to the medical office for guideline-compliant pegfilgrastim administration. The CONVENIENCE study aimed to evaluate patient, nurse, and physician preferences as well as health economics for pegfilgrastim administration either with OBI or manually using a pre-filled syringe (PS).

**Methods:**

Patients with early breast cancer, receiving two or three weekly anthracycline/cyclophosphamide or three weekly taxane-based CTx, and patients with Non-Hodgkin lymphoma (NHL) receiving first-line R-CHOP-14 or -21 were randomized 1:1 to receive both pegfilgrastim application forms for four consecutive CTx cycles in an alternating sequence starting either with OBI or PS. Primary endpoint was patient preference, assessed by questionnaires.

**Results:**

A total of 308 patients were evaluable in the per-protocol analysis. Patients slightly preferred OBI over PS (OBI, *n* = 133, 43.2%; vs. PS, *n* = 111, 36.0%; *p*-value = 0.159), while study nurses slightly preferred PS (*n* = 19, 46.3%) over OBI (*n* = 18, 43.9%) and physicians clearly preferred PS (*n* = 24, 58.8%) over OBI (*n* = 15, 36.6%). Among patients with preference for OBI, saving of time was their major reason for preference (53.4%). Pegfilgrastim was administered 24–72 h after each CTx cycle in 97.6% of OBI and 63.1% of PS applications.

**Conclusion:**

The OBI was slightly preferred by patients and saving time was the major reason for their preference. PS was physicians’ most preferable choice and slightly preferred by nurses. Using OBI, pegfilgrastim was almost always administered within the time period recommended by current guidelines, while it was often not applied as specified using PS.

**Trial registration:**

No: ClinicalTrials.gov No. NCT03619993. Registered on June 25, 2018

## Introduction

Neutropenia is among the most frequently observed side effects of myelosuppressive chemotherapy in patients with cancer [1]. Especially in the presence of fever (i.e., febrile neutropenia, FN), neutropenia is associated with increased infection-related morbidity and mortality [2, 3]. Limiting the doses of chemotherapy that can be tolerated, FN may lead to treatment delays, dose reductions, and/or chemotherapy discontinuations, associated with an increased demand of healthcare resources and poorer outcomes in several cancer types, including breast cancer and Non-Hodgkin’s lymphoma (NHL) [1, 4, 5]. Prophylaxis with recombinant granulocyte colony stimulating growth factors (G-CSFs), particularly its pegylated form, has been shown to reduce incidence, severity, and duration of FN in patients with solid tumors or lymphoma [6, 7], and to reduce the likelihood of chemotherapy dose reductions and the number of hospitalizations due to FN [8, 9]. Accordingly, the German S3 guidelines as well as ASCO (American Society of Clinical Oncology) and ESMO (European Society for Medical Oncology) clinical practice guidelines recommend recombinant G-CSF, such as pegfilgrastim, as primary prophylaxis for cancer patients who have a high risk for developing FN based on treatment-, disease-, and patient-related factors [10–12].

Pegfilgrastim (Neulasta®; AMGEN Inc., Thousand Oaks, CA, USA), approved for the reduction of neutropenia and the incidence of FN in adult patients treated with cytotoxic chemotherapy (CTx) for malignancy in the year 2002 [13], should be administered at least 24 h after CTx as per current Summary of Product Characteristics of Neulasta® [14]. Optimal timing is important since simultaneous administration of G-CSFs and chemotherapeutic agents carries the risk of enhanced myelosuppression and may subsequently even increase the incidence of neutropenic complications [15, 16]. Next-day pegfilgrastim application, however, involves logistic issues and has been considered inconvenient for some patients and physicians, as patients either may have to return to the clinic or medical office the day after chemotherapy to receive pegfilgrastim application [17] or may have it administered by themselves or by another person at home, which might involve application issues. A pooled analysis of two large US healthcare claims databases demonstrated that with 13.4% a minority, but considerable proportion of patients receive pegfilgrastim prophylaxis on the same day as chemotherapy [18]. To address logistic issues and the requirement of return visits to the medical office, an alternative pegfilgrastim application form was introduced in 2014 [19]. The on-body injector (OBI) (Onpro®; AMGEN Inc., Thousand Oaks, CA, USA), co-packaged with a single pre-filled syringe, is designed to be applied on patient’s abdomen or arm on the same day as chemotherapy and to deliver pegfilgrastim automatically the next day, approximately 27 h after activation [14]. As such, it might optimize pegfilgrastim prophylaxis by improving the timepoint of administration and saving time and costs both for patients and healthcare professionals (HCPs). The CONVENIENCE study was designed to evaluate patient, nurse, and physician preferences and health economics for pegfilgrastim administration in Germany either manually using a pre-filled syringe (PS) or automatically using the OBI.

## Methods

### Study population

Patients aged ≥ 18 years, diagnosed with early breast cancer (EBC) planned to receive two or three weekly anthracycline/cyclophosphamide or three weekly taxane-based chemotherapy or NHL patients planned to receive first-line rituximab, cyclophosphamide, doxorubicin hydrochloride, vincristine and prednisone (R-CHOP)-14 or R-CHOP-21 immunochemotherapy with the indication for G-CSF prophylaxis with pegfilgrastim, were eligible. Furthermore, patients had to be able to read and understand German, have an Eastern Cooperative Oncology Group (ECOG) performance status of ≤ 2, a life expectancy of  > 3 months, an absolute neutrophil count ≥ 1.5 × 10^9^, and had to be without G-CSF support prior to randomization. Key exclusion criteria included hypersensitivity to the active substance or to any of the excipients, acute infections, and prior bone marrow or stem cell transplants. All patients provided written informed consent.

### Study design

**Fig. 1:**
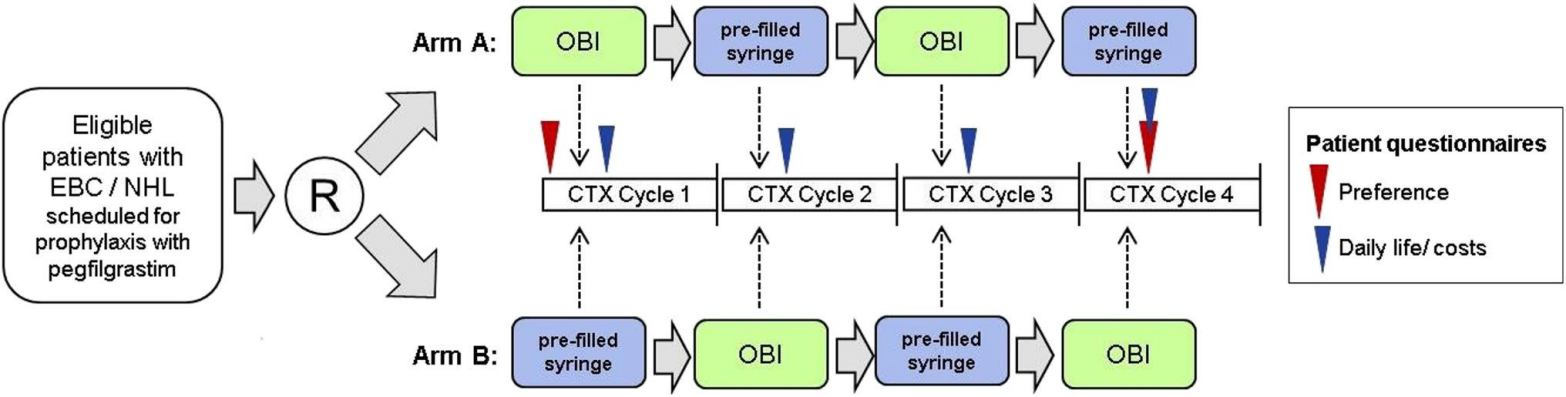
Overall study design. CTX chemotherapy, EBC early breast cancer, NHL Non-Hodgkin lymphoma, OBI on-body injector for pegfilgrastim, R randomization. Patient questionnaires: The questionnaire on patient preference had to be completed by the patient at baseline, prior to the first administration of pegfilgrastim and at the individual end of study after the fourth or last application of pegfilgrastim. The questionnaire on patient’s daily life and cost relevant factors had to be completed by the patient on day 2–4 of each cycle

CONVENIENCE was an open-label, randomized, two-arm, controlled cross-over study according to §23b MPG (Clinical Trials.gov No. NCT03619993) designed to assess patient preference for pegfilgrastim administration via OBI compared to manual injection via PS (Fig. 1). At 41 study sites in Germany (mainly outpatient oncological practices), patients were to be observed for 4 consecutive cycles of chemotherapy supported with both pegfilgrastim application forms (PS or OBI) in an alternating sequence either starting with OBI (arm A, OBI-PS-OBI-PS) or manual injection (arm B, PS-OBI-PS-OBI). Prior to the first chemotherapeutic treatment supported by G-CSF prophylaxis with pegfilgrastim, patients were 1:1 randomized to arm A or arm B via permuted block randomization stratified by tumor entity. Pegfilgrastim was given at 6-mg solution for manual injection (PS; 0.6 ml) as per current recommendations for administration, or subcutaneous injection via OBI (0.64 ml co-packed pre-filled syringe). In this study, the OBI always had to be applied at the outpatient oncological practice or hospital where the patient had received their chemotherapeutic treatment. PS administration was done as per clinical routine and could be administered at either the outpatient oncological practice or hospital or at the patient’s family practice by a general practitioner. Furthermore, patients could receive the PS at their home, administered by themselves, or caregiver-assisted. Regular end of study was defined as the timepoint when the last patient had returned their second patient-preference questionnaire or 4 weeks after the last patient had received their last pegfilgrastim application within the study, whatever came first.

### Study endpoints

The primary endpoint of the study was to evaluate patients’ preference for either of the two pegfilgrastim application forms (OBI or PS) after the individual end of study (EOS). Secondary endpoints included the actual pegfilgrastim application timepoint, reasons for patients’ preference, influence of either application form on daily life of the patient, preference of the study nurse and treating physician and cost-related factors of either application form.

### Questionnaires

Patients’ preference for either pegfilgrastim application form (primary endpoint) was assessed by patient preference questionnaires, completed by the patient at baseline and at the individual EOS. Patients were asked to select either PS or OBI or no preference. Secondary endpoints, including influence of either pegfilgrastim form on patient’s daily life and cost-relevant factors, as well as treating physician’s and study nurse’s preference for either pegfilgrastim application were assessed by questionnaires.

### Statistical analysis

Assuming a drop-out rate of 10%, an estimated sample size of *n* = 400 patients was required to reject the null hypothesis with a power of 90% and an alpha error of 5%.

Analysis of the primary endpoint was conducted based on the per-protocol set (PPROT), including patients who met all inclusion/exclusion criteria and who had received both application forms twice each in an alternating sequence during four consecutive cycles of chemotherapy as assigned per study protocol and had completed the second preference questionnaire after the individual EOS. Patient preference was evaluated for the PPROT population overall, and stratified by baseline characteristics, by tumor entity, by location where the manual injection had been applied, and by the distance between medical office and patients’ residence. To test the significance of difference in patient preference between the two administration forms (primary endpoint), McNemar’s Test was used. Analyses of secondary endpoints were performed using descriptive statistics, based on either the PPROT or study center (SC) population, the latter comprising all study centers in which pegfilgrastim had been applied at least once in each of the two application forms.

## Results

Between June 2018 and June 2019, 404 patients were enrolled in 41 study centers across Germany, of whom 402 patients were randomly assigned to start pegfilgrastim prophylaxis either with OBI (arm A, *n* = 201) or PS (arm B, *n* = 201).

**Fig. 2:**
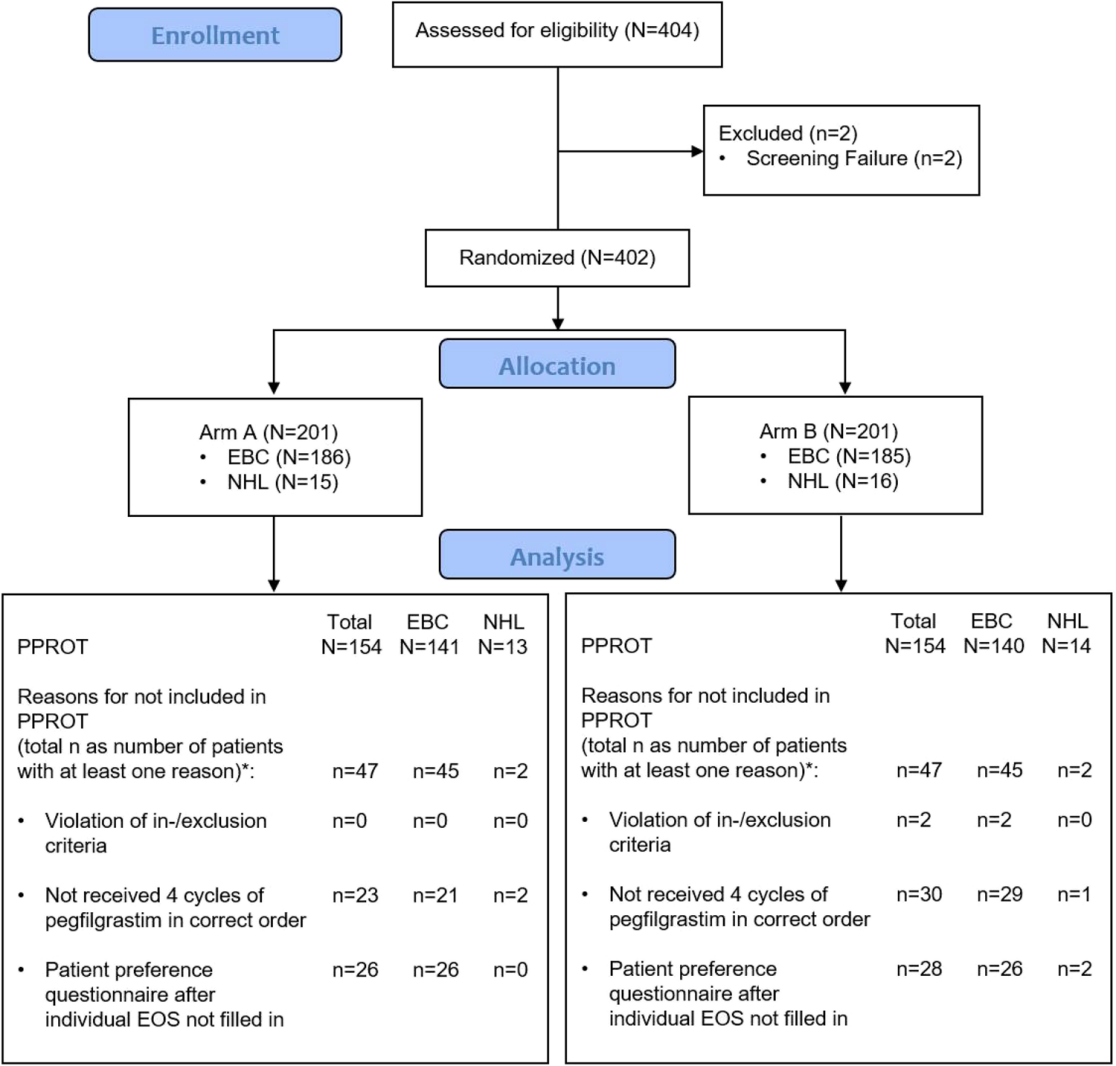
CONSORT flow diagram. EBC early breast cancer, EOS end of study, N/n number, NHL Non-Hodgkin lymphoma; PPROT per-protocol set; *Numbers of the listed reasons do not necessarily sum up to the total number of reasons for exclusion from PPROT as for some patients, more than one reason was applicable

Patient preference questionnaires were returned from 368 patients (93.2%) prior to start of the study and from 353 patients (91.9%) at the EOS. The return rate of the secondary endpoint patient questionnaire on daily life and costs was 92%. Return rates of both study nurse’s and physician’s preference questionnaires at study start were 95.1% each and at the EOS 97.6% each (data not shown). The following results are only presented for the PPROT population (*n* = 308; Fig. 2) and for the SC population (*n* = 41).

With respect to the location of manual injection (PS), most patients had received their PS at home (private environment, *n* = 179; 60.5%), while 68 (23.0%) patients had received it at an oncological practice and 15 (5.1%) patients at their general practitioner (family practice). Other patients (*n* = 34; 11.5%) had received their PS at mixed locations (i.e., the two manual injections at different locations).

### Patient characteristics

Median age of the patients was 55 years (range 29–85) and 57 years (range 28–83) in patients of arm A and arm B, respectively. Most patients (*n* = 146, 94.8% in arm A; *n* = 143, 92.9% in arm B) were female and had an ECOG status of 0 (arm A, *n* = 127, 82.5%; arm B, *n* = 118, 76.6%). A majority of 281 patients (arm A, *n* = 141, 91.6%; arm B, *n* = 140, 90.9%) were diagnosed with breast cancer and only 27 patients with NHL (arm A, *n* = 13, 8.4%; arm B, *n* = 14, 9.1%). The most frequently observed chemotherapy regimen among breast and NHL cancer patients was anthracycline/cyclophosphamide-based and R-CHOP-21, respectively. Overall, patient characteristics were well balanced between the study arms (Table 1).

**Table 1:** Patient characteristics at baseline (PPROT population)

Characteristic	Total (*N* = 308)	Arm A (*N* = 154)	Arm B (*N* = 154)
Age at date of informed consent (years)			
Median (min–max)	56 (28–85)	55 (29–85)	57 (28–83)
Sex, *n* (%)			
Female	289 (93.8%)	146 (94.8%)	143 (92.9%)
Male	19 (6.2%)	8 (5.2%)	11 (7.1%)
Performance status at baseline, *n* (%)			
ECOG 0	245 (79.5%)	127 (82.5%)	118 (76.6%)
ECOG 1	63 (20.5%)	27 (17.5%)	36 (23.4%)
Tumor entity, *n* (%)			
Early breast cancer	281 (91.2%)	141 (91.6%)	140 (90.9%)
Non-Hodgkin Lymphoma	27 (8.8%)	13 (8.4%)	14 (9.1%)
Early breast cancer	*N* = 281	*N* = 141	*N* = 140
Treatment regimen, *n* (%)			
Anthracycline/cyclophosphamide-based	228 (81.1%)	115 (81.6%)	113 (80.7%)
Taxane-based	31 (11.0%)	13 (9.2%)	18 (12.9%)
Anthracycline-based	16 (5.7%)	9 (6.4%)	7 (5.0%)
Anthracycline/cyclophosphamide/taxane-based	6 (2.1%)	4 (2.8%)	2 (1.4%)
Other	-	-	-
Non-Hodgkin lymphoma	*N* = 27	*N* = 13	*N* = 14
Type of lymphoma, *n* (%)			
B-cell lymphoma	26 (96.3%)	12 (92.3%)	14 (100%)
Treatment regimen, *n* (%)			
R-CHOP-21	17 (63.0%)	7 (53.8%)	10 (71.4%)
R-CHOP-14	9 (33.3%)	5 (38.5%)	4 (28.6%)
Other	1 (3.7%)	1 (7.7%)	-
Distance between medical office and patient residence (km), *n* (%)			
Distance ≤ 5 km	74 (24.0%)	42 (27.3%)	32 (20.8%)
5 km > distance ≤ 10 km	50 (16.2%)	27 (17.5%)	23 (14.9%)
10 km > distance ≤ 20 km	60 (19.5%)	29 (18.8%)	31 (20.1%)
20 km > distance ≤ 50 km	106 (34.4%)	49 (31.8%)	57 (37.0%)
Distance > 50 km	18 (5.8%)	7 (4.5%)	11 (7.1%)

Percentages refer to total *N*, or if specified otherwise, to the number of patients with early breast cancer or Non-Hodgkin lymphoma

*ECOG*, Eastern Cooperative Oncology Group; *N/n*, number; *PPROT*, per protocol set; *R-CHOP*, rituximab-cyclophosphamide-doxorubicin hydrochloride-vincristine-prednisone

### Patient preference (primary endpoint)

**Fig. 3:**
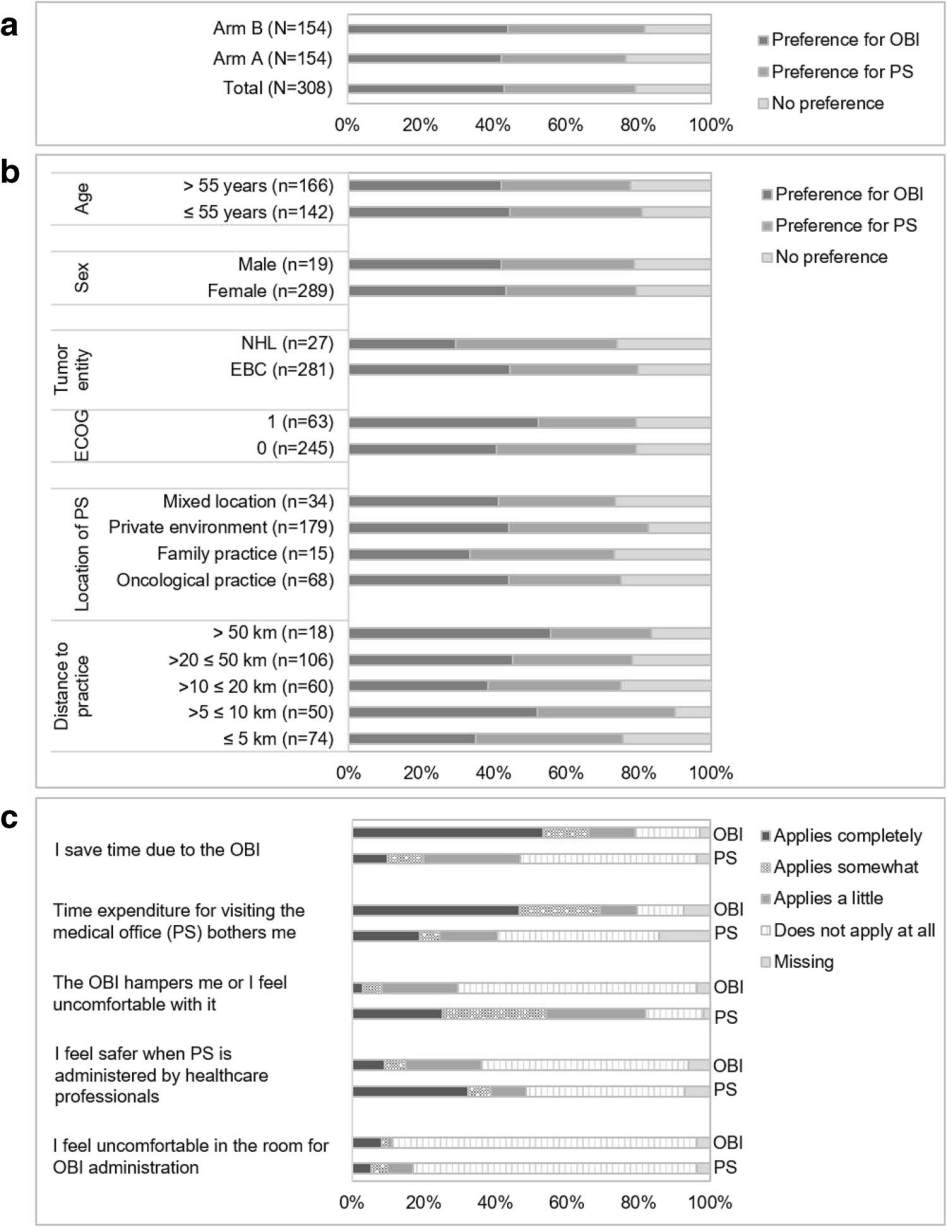
Patient preferences and reasons for their preference at the end of study. **a** Patient preference by study arms. **b** Patient preference stratified by subgroups. **c** Reasons for patient preference stratified by patient preference. OBI on-body injector for pegfilgrastim, PS pre-filled syringe

As illustrated in Fig. 3a, there was a slight tendency towards a patient preference for OBI over PS (OBI, *n* = 133, 43.2%; PS, *n* = 111, 36.0%; *p*-value = 0.159) after the individual EOS. A similar tendency was observed in both study arms (arm A, *n* = 65, 42.2% OBI vs. *n* = 53, 34.4% PS; arm B, *n* = 68, 44.2% OBI vs. *n* = 58, 37.7% PS). Since there were no marked differences in overall study results between the study arms, the following results will be presented for the total patient population only.

#### Patient preference stratified by subgroups

Subgroup analyses of patient preference are depicted in Fig. 3b. No notable differences in preference by age or sex (94% of the patients were females) were observed. In contrast to EBC patients, who preferred OBI over PS (OBI, *n* = 125, 44.5%; PS, *n* = 99, 35.2%), patients with NHL preferred PS; however, the group size was small (OBI, *n* = 8, 29.6%; PS, *n* = 12, 44.4%). Compared to patients with an ECOG status of 0 (OBI, *n* = 100, 40.8%; PS, *n* = 94, 38.4%), preference for OBI was more pronounced in patients with an ECOG status of 1 (OBI, *n* = 33, 52.4%; PS, *n* = 17, 27.0%). Furthermore, the proportions of patients preferring OBI over PS were higher in subgroups of patients having received all manual injections in an oncological practice (OBI, *n* = 30, 44.1%; PS, *n* = 21, 30.9%), in a private environment (OBI, *n* = 79, 44.1%; PS, *n* = 69, 38.5%) and in mixed locations (OBI, *n* = 14, 41.2%; PS, *n* = 11, 32.4%). In contrast, more patients who had received manual injections at a family practice preferred PS over OBI (OBI, *n* = 5, 33.3%; PS, *n* = 6, 40.0%). Regarding to the distance between the medical office and patient’s residence, the proportion of patients preferring PS was higher than those preferring OBI in the subgroup of patients having a distance of ≤ 5 km (OBI, *n* = 26, 35.1%; PS, *n* = 30, 40.5%), whereas in the subgroups of patients having a distance > 5 km, a greater proportion of patients preferred OBI over PS, including the subgroups of patients with > 5 ≤ 10 km (OBI, *n* = 26, 52.0%; PS, *n* = 19, 38.0%), > 10 ≤ 20 km (OBI, *n* = 23, 38.3%; PS, *n* = 22, 36.7%), > 20 ≤ 50 km (OBI, *n* = 48, 45.3%; PS, *n* = 35, 33.0%), and ≥ 50 (OBI, *n* = 10, 55.6%; PS, *n* = 5, 27.8%).

#### Reasons for preference

Among the reasons for patients preferring OBI over PS (Fig. 3c), saving time due to the OBI and the time expenditure for visiting the medical office for PS administration were the reasons that mostly applied to them (*n* = 71, 53.4%, and *n* = 62, 46.6%, respectively), while in the subgroup of patients with preference for PS, these reasons applied completely to a relatively lower proportion of patients (*n* = 11, 9.9%, and *n* = 21, 18.9%, respectively). In contrast, patients preferring PS more often indicated to feel uncomfortable with the OBI and to feel safer when pegfilgrastim was administered by HCPs (*n* = 28, 25.2%, and *n* = 36, 32.4%, respectively), as compared to patients with preference for OBI (*n* = 4, 3.0%, and *n* = 12, 9.0%).

### Time between the end of chemotherapy and start of pegfilgrastim application

**Fig. 4:**
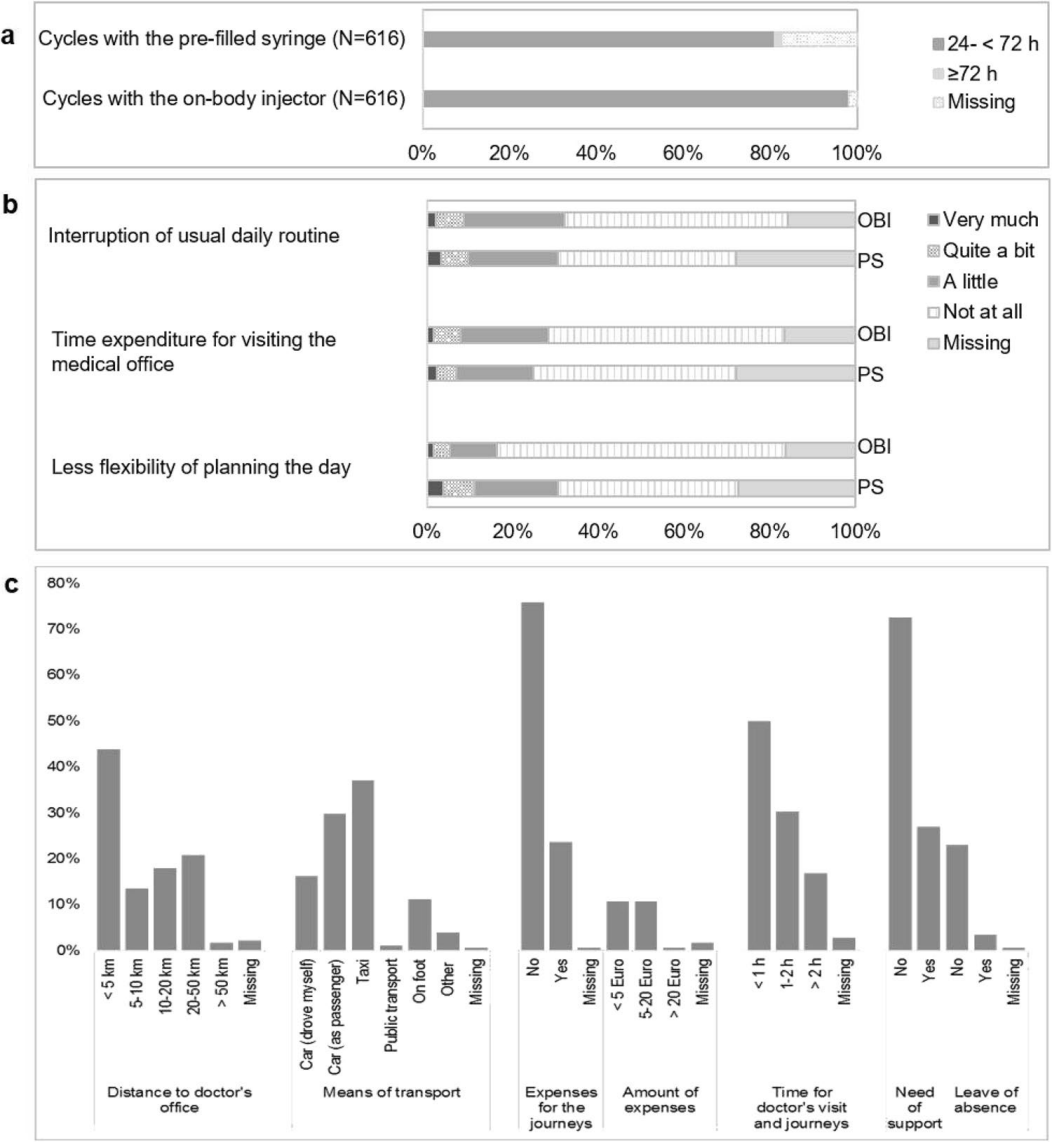
Application timepoint, influence on patients’ daily life, and costs. **a** Time between end of chemotherapy and start of pegfilgrastim application. **b** Influence of the pegfilgrastim application on patients’ daily life stratified by OBI- or PS-supported cycles. **c** Cost-related factors (PS-supported cycles only)

Administration timepoint of the OBI (see Fig. 4a) was calculated by adding 27 h to the timepoint at which the OBI had been applied. Pegfilgrastim was administered 24–48 h after chemotherapy in 97.6% (*n* = 601) of all OBI-supported cycles (*n* = 616), and in 62.0% (*n* = 382) of all PS-supported cycles (*n* = 616). In 22.1% (*n* = 136) of PS cycles, pegfilgrastim was applied < 24 h and in 1.1% (*n* = 7) cycles each 48–72 h and ≥ 72 h. With the OBI, pegfilgrastim was administered < 24 h in 0.5% of cycles (*n* = 3).

### Influence on daily life and cost-related factors

#### Influence on daily life

Evaluating OBI-supported cycles only, patients reported more often that their daily routine and social life was not interrupted at all (*n* = 321, 52.1%, and *n* = 339, 55.0%) as compared to PS-supported cycles (*n* = 256, 41.6%, and *n* = 291, 47.2%). Furthermore, patients reported in 67.2% (*n* = 414) of OBI-supported cycles that the pegfilgrastim application did not lead to any reduced flexibility of planning of the day, which was observed in a clearly lower frequency of PS cycles (*n* = 259, 42.0%) (Fig. 4b).

#### Cost-related factors (PS-supported cycles only)

The time spent for both the journey to the medical office and the doctor’s visit in PS-supported cycles away from home was reported to be ≤ 1 h in 50% of the cycles (*n* = 89), while it was reported to be 1–2 h in 30.3% (*n* = 54) and > 2 h in 16.9% (*n* = 30) of cycles. In most of the cycles (*n* = 121, 68.0%), patients needed another person to drive them; thereof, a taxi was taken in 37.1% (*n* = 66). Patients reported in most cycles (*n* = 135, 75.8%) that they had no expenses for journeys to and from the medical office and that they were not in need of any support in 72.5% (*n* = 129) (please see Fig. 4c for further details).

### Study nurse and physician preference

**Fig. 5:**
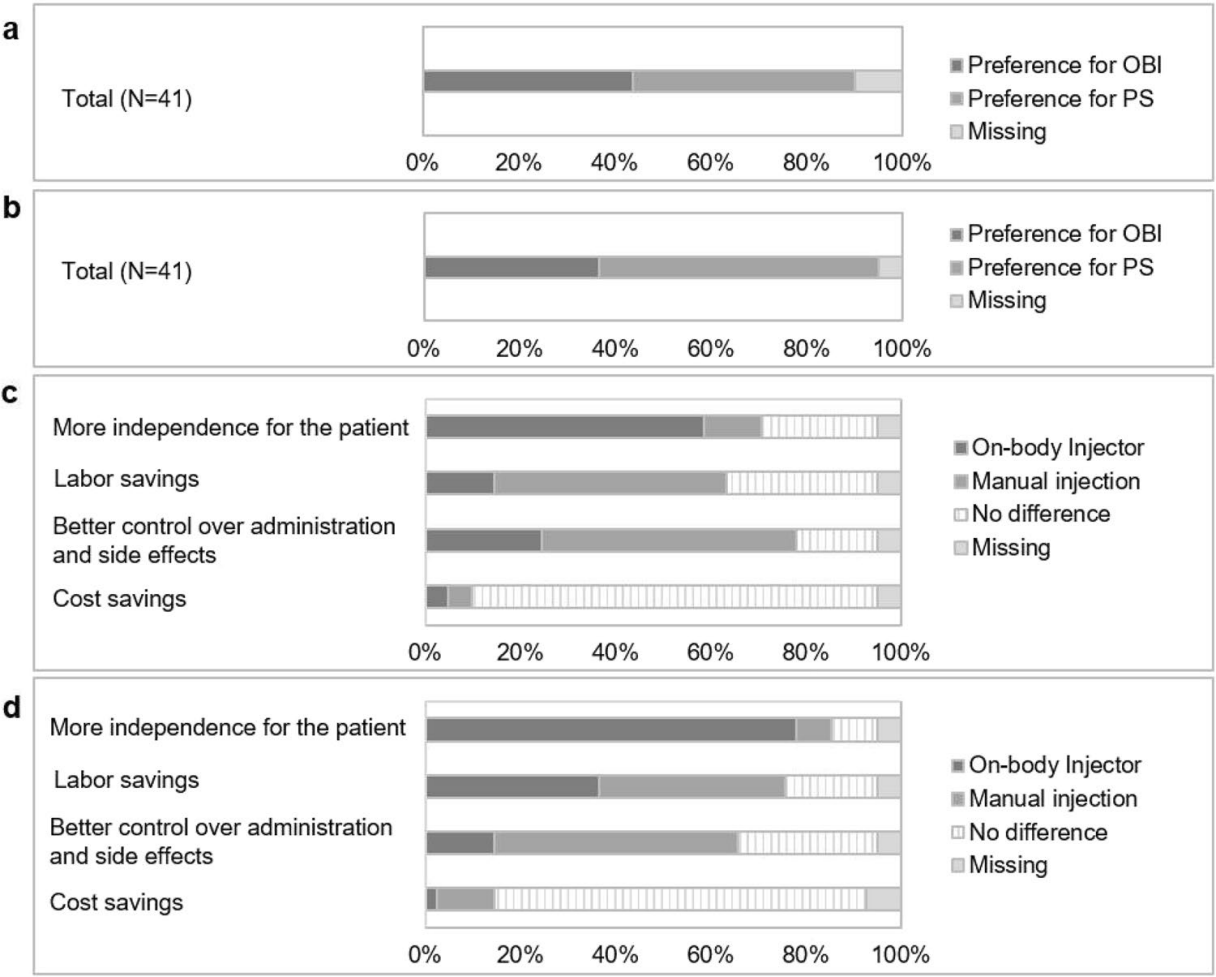
Study nurse and physician preferences and reasons for their preference at the end of study. **a** Study nurse preference. **b** Physician preference. **c** Reasons for preference of the study nurse. **d** Reasons for preference of the physician. OBI on-body injector for pegfilgrastim; PS pre-filled syringe

Findings of the analyses on study nurses’ and physicians’ preferences as well as reasons for their preference are depicted in Fig. 5. At EOS, the proportion of study nurses preferring PS (*n* = 19, 46.3%) was slightly higher as compared to those preferring OBI (*n* = 18, 43.9%). Among physicians, the difference between preferences for PS (*n* = 24, 58.8%) and OBI (*n* = 15, 36.6%) was more pronounced.

#### Reasons for preference

A markedly higher proportion of study nurses selected the OBI as the application form providing more independence for the patient as compared to those who selected PS (OBI, *n* = 24, 58.5%, vs. PS, *n* = 12, 12.2%). More study nurses selected PS than OBI with regard to the questions which application form rather yields labor savings at the medical office (PS, *n* = 20, 48.8%, vs. OBI, *n* = 6, 14.6%) and which application form provides a better control over administration and side effects (PS, *n* = 22, 53.7%, vs. OBI, *n* = 10, 24.4%). There was a clear difference in proportions of physicians who selected OBI as the application form providing more independence for the patient (*n* = 32, 78.0%) as compared to those who selected PS regarding this question (*n* = 3, 7.3%). With regard to both the questions which application form rather yields labor savings at the medical office and which application form provides a better control over administration and side effects, more physicians selected PS than OBI (PS, *n* = 16, 39.0%, vs. OBI, *n* = 15, 36.6%, and PS, *n* = 21, 52.1%, vs. *n* = 6, 14.6%).

## Discussion

The CONVENIENCE study was designed to evaluate patient, nurse, and physician preferences as well as health economics for pegfilgrastim administration with either PS or OBI. Return rates of all questionnaires were very high (≥ 90 to 100%), which was key to the success of the study as all study objectives were exclusively or partly assessed by questionnaires. At the EOS, it was found that patients slightly preferred OBI over PS (43.2% OBI vs. 36.0% PS). However, the difference in proportions of patients was not statistically significant. Both study arms showed the same tendency, indicating that patients’ preference was independent of the sequence of application.

The different locations where patients could receive their administrations with PS reflect the structure of the German Healthcare System, where also a general practitioner could administer the PS (family practice). This may not be feasible in other countries. In a study from the US investigating clinical practice of pegfilgrastim administration, it was reported that the majority (67.0%) of pegfilgrastim injections were administered in an outpatient setting (office, clinic, infusion center), while 13.8% were administered in a hospital and 18.4% at patients’ home either by themselves or caregiver-assisted [17].

In the present study, the number of patients having received all PS administrations in a private environment (*n* = 179; 60.5%) was by far higher compared to the number of patients with all PS administrations at an oncological practice (*n* = 68; 23.0%) or at a family practice (*n* = 15; 5.1%), which, especially for the latter subgroup, limits the interpretability of the data and might have influenced patients’ preference; as for those patients having received PS in a private environment, the OBI may not have offered major time or cost advantages. Nevertheless, subgroup analyses showed that even among patients having received PS in a private environment, a slight majority had chosen the OBI as preferred option, possibly as it eliminates the need to plan and administer manual injection and thus provides greater independency to the patient. This is further supported by the finding that patients’ daily life had been interrupted to a lesser extent in cycles with the OBI only as compared to cycles with PS only.

Patients with preference for OBI rated saving of time as major reason for their preference, although this applied completely to a relatively lower proportion as one may have expected (53.4%). This may be attributed to the above-outlined fact that most patients had received PS in a private environment and furthermore to the fact that the majority lived relatively close to the medical office. Of note, with increasing distance between medical office and patients’ residence, the proportion of patients with preference for OBI tended to be higher than the proportion of patients preferring PS, suggesting that especially these patients might benefit most from the OBI in terms of saving time and costs.

While age did not play a major role in patients’ decision for either of the two application forms, the ECOG status at baseline had an influence, insofar as patients having an ECOG status of 0 had no clear preference, while for patients having an ECOG status of 1, the OBI seemed to be the better option, possibly as for this group of patients, return visits to the medical office might be particularly strenuous. This would be in line with findings of a cross-sectional survey on US patients’ and physicians’ preferences for pegfilgrastim application forms [20], in which physicians indicated to more likely prescribe the OBI when the patient’s health was particularly compromised. At the same time, however, it was also suggested that especially for patients in a poor condition, an additional appointment at the medical office might be reasonable as it offers the opportunity to more closely monitor the patient’s condition [20]. The finding of the present study that higher proportions of both study nurses and physicians selected PS as the application form providing a better control over administration and side-effects might support this assumption and might have influenced their preference the most at the EOS, as a higher proportion of both study nurses and physicians indicated to prefer PS over OBI.

Patients with preference for PS more often indicated that they felt uncomfortable with the OBI and felt safer when pegfilgrastim was administered by HCPs, which is consistent with findings of the aforementioned US survey [20], in which patients with preference for in-clinic pegfilgrastim administrations indicated to prefer professional staff to administer the medication. Although successful delivery rates by the OBI have previously been demonstrated [19], these findings suggest that some patients may still have reservations about delivery of pegfilgrastim in the absence of professional staff. Once they have gained experience with the OBI, however, it seems to be more likely that patients choose this application form again as observed by Hauber et al. [20].

There was a markedly higher compliance with current guidelines for timepoint of pegfilgrastim application within this study in OBI-supported cycles (97.6%) as compared to PS-supported cycles overall (62.0%), and particularly, as compared to cycles with PS at an oncological practice (40.9%). This is important as the effectiveness of G-CSFs like pegfilgrastim depends on the optimal timing [14, 15], although it has to be noted that neither correct medication delivery through OBI nor reasons for non-adherence were captured in this study and it can only be assumed that pegfilgrastim was delivered 27 h after activation without issues as correct performance of OBI had been demonstrated before [19]. In 0.5% of OBI-supported cycles, pegfilgrastim had been administered within 24 h after CTx potentially due to logistics or resource situation at the medical office.

Overall, the results of the CONVENIENCE study demonstrate that the choice for an application form needs to be made under consideration of multifactorial aspects for each patient individually. The data show a tendency, suggesting that patients might more likely profit from the OBI when giving a high priority in time saving and independence, while PS might be the better option for patients, either for whom the OBI does not offer much time saving, or for whom a close support by HCP is important. The benefits of saving an additional burdensome return visit to the medical office using the OBI need to be weighed against the need of close monitoring of patients in poor health conditions.

When interpreting the results, it has to be kept in mind that only a minority of the patients (*n* = 19) were males and a rather low number of patients with NHL were included into the study (*n* = 27) which limits the interpretability of the data within these subgroups. The same applies to the subgroup of patients with all manual injections at a family practice (*n* = 15). Notwithstanding the above, the overall analytical study population, in which only patients who had been observed for four consecutive CTx cycles and supported by both application forms twice each in the correct order were considered, provided solid data to evaluate patients’ preference at the EOS which was the primary study objective. Furthermore, the crossover design of the study served to exclude potential bias which might have been generated when all patients had started with the same application form.

### Main conclusions of the study

In conclusion, the OBI was slightly preferred by patients and saving time was the major reason for their preference. PS was slightly preferred by study nurses, while physicians had a clear preference for PS over OBI. Pegfilgrastim was almost always applied within the recommended time period when using the OBI, while it was not always applied as recommended when it was manually administered.

## Data Availability

Clinical data were documented in electronic Case Report Forms (eCRFs; *iostudy office edc*, iOMEDICO) and are the property of iOMEDICO. The data, including the outcomes of the paper-based questionnaires, are not publicly available.

## Abbreviations

ASCO: American Society of Clinical Oncology
CTx: Chemotherapy
EBC: Early breast cancer;
ECOG: Eastern Cooperative Oncology Group
EOS: End of study
ESMO: European Society for Medical Oncology
FN: Febrile neutropenia
G-CSF: Granulocyte colony stimulating growth factor
HCPs: Healthcare professionals
NHL: Non-Hodgkin lymphoma
OBI: On-body injector
PPROT: Per-protocol set
PS: Pre-filled syringe
R-CHOP: Rituximab-cyclophosphamide-doxorubicin hydrochloride-vincristine-prednisone
SC: Study center
